# Chitin Degradation Machinery and Secondary Metabolite Profiles in the Marine Bacterium *Pseudoalteromonas rubra* S4059

**DOI:** 10.3390/md19020108

**Published:** 2021-02-12

**Authors:** Xiyan Wang, Thomas Isbrandt, Mikael Lenz Strube, Sara Skøtt Paulsen, Maike Wennekers Nielsen, Yannick Buijs, Erwin M. Schoof, Thomas Ostenfeld Larsen, Lone Gram, Sheng-Da Zhang

**Affiliations:** Department of Bioengineering, Technical University of Denmark, DK-2800 Kgs Lyngby, Denmark; xwan@dtu.dk (X.W.); tispe@bio.dtu.dk (T.I.); milst@dtu.dk (M.L.S.); ssp@sbtinstruments.com (S.S.P.); mweni@dtu.dk (M.W.N.); yabu@dtu.dk (Y.B.); erws@dtu.dk (E.M.S.); tol@bio.dtu.dk (T.O.L.); gram@bio.dtu.dk (L.G.)

**Keywords:** chitin, chitinase, chitin degradation machinery, *Pseudoalteromonas*, secondary metabolites

## Abstract

Genome mining of pigmented *Pseudoalteromonas* has revealed a large potential for the production of bioactive compounds and hydrolytic enzymes. The purpose of the present study was to explore this bioactivity potential in a potent antibiotic and enzyme producer, *Pseudoalteromonas rubra* strain S4059. Proteomic analyses (data are available via ProteomeXchange with identifier PXD023249) indicated that a highly efficient chitin degradation machinery was present in the red-pigmented *P. rubra* S4059 when grown on chitin. Four GH18 chitinases and two GH20 hexosaminidases were significantly upregulated under these conditions. GH19 chitinases, which are not common in bacteria, are consistently found in pigmented *Pseudoalteromonas,* and in S4059, GH19 was only detected when the bacterium was grown on chitin. To explore the possible role of GH19 in pigmented *Pseudoalteromonas*, we developed a protocol for genetic manipulation of S4059 and deleted the GH19 chitinase, and compared phenotypes of the mutant and wild type. However, none of the chitin degrading ability, secondary metabolite profile, or biofilm-forming capacity was affected by GH19 deletion. In conclusion, we developed a genetic manipulation protocol that can be used to unravel the bioactive potential of pigmented pseudoalteromonads. An efficient chitinolytic enzyme cocktail was identified in S4059, suggesting that this strain could be a candidate with industrial potential.

## 1. Introduction

Marine microorganisms have emerged as a promising source of novel antimicrobial compounds or hydrolytic enzymes [1,2]. In particular, the marine genus *Pseudoalteromonas* harbors a wide range of bioactive compounds with antimicrobial, antifouling, and algicidal activities [3,4,5,6]. Based on both phenotypes and genome-wide analyses, *Pseudoalteromonas* can be divided into two groups: pigmented and non-pigmented species [3]. The genomes of pigmented species contain a high number of biosynthetic gene clusters (BGCs) as compared to those of non-pigmented species [7]. Both groups carry the genetic potential to produce a wide range of glycosyl hydrolases, and the pigmented *Pseudoalteromonas* especially harbors a powerful chitin degrading machinery containing several chitinolytic enzymes [7].

Chitin is the most abundant carbon source in the marine environment [8], where it is present in three crystalline allomorphs: α-, β-, and γ-chitin. α-chitin has antiparallel chains, β-chitin has parallel chains, and γ-chitin has the mixture of both chains [9]. Chitin in nature is predominantly degraded by microorganisms [10], and chitin degradation depends on secreted extracellular chitinases (EC.3.2.1.14) and other chitinolytic enzymes/proteins, such as lytic polysaccharide monooxygenases (LPMOs) [11]. Chitinases are glycoside hydrolases (GHs) and are classified into GH18, GH19, and GH20 in the CAZy database [12,13]. The GH18 family chitinases are common in bacteria, whereas the GH19 chitinases are mostly found in plants and are believed to function as a defense mechanism against fungal pathogens [14,15]. Chitinases of both families catalyze the degradation of chitin polymers [12]. The GH 20 family β-N-acetylhexosaminidases hydrolyze amorphous chitin polymers [16], and the LPMOs are metalloenzymes that cleave glycosidic bonds in crystalline chitin and facilitate access of chitinase [17]. Paulsen et al. [7] found that pigmented and non-pigmented *Pseudoalteromonas* evolved divergent GH profiles in their genomic contents. Further, all pigmented *Pseudoalteromonas* species contain at least one GH19 chitinase, which is rarely reported in bacteria. However, only a very few non-pigmented *Pseudoalteromonase* species contain a GH19 chitinase [7]. A GH19 chitinase of the pigmented *Pseudoalteromonas tunicata* CCUG 44952T has been heterologously expressed in *Escherichia coli* and displayed antifungal activity [18]. However, whether this is the dominant role of GH19 in pigmented *Pseodoalteromonas* is yet to be investigated.

The secondary metabolome of several bacteria is influenced by carbon-source, and chitin may serve to enhance the production of secondary metabolites, as observed in strains of Vibrionaceae [19,20,21]. Likewise, the addition of chitin to *Streptomyces coelicolor* A3 (2) growing in autoclaved soil induced the expression of genes associated with secondary metabolites’ production [22]. Due to the potent chitinolytic machinery in *Pseudoalteromonas* [7], we speculated that there could be a link between chitin degradation and secondary metabolism. Since *P. rubra* S4059 dedicates 15% of its genome to secondary metabolites [7] and as other pigmented pseudoalteromonads contain GH19 chitinases [7], we further explored the bioactivity of this prodigiosin-producing strain as a model organism. The purpose of this study was to explore the chitin degradation machinery and secondary metabolite profiles when grown on chitin and to investigate the possible function of GH19 chitinase in *P. rubra* S4059.

## 2. Results

### 2.1. In Silico Analysis of Chitin Degrading Machinery and Bioactive Potential in P. rubra S4059

The genome of S4059 consists of two circular chromosomes, of 4,595,233 bp and 1,348,119 bp, with an average G + C content of 47.71% and 46.93%. Fourteen putative chitinolytic enzymes were identified according to the prediction of the S4059 proteome in Uniprot (proteome ID UP000305729), including seven of the GH18 chitinase family, two of the GH19 chitinase family, and three of the GH20 hexosaminidase family, as well as two lytic polysaccharide monooxygenases (LPMOs) of the auxiliary activity (AA) family 10 (Table 1). All the chitinolytic enzymes contained a signal peptide at the N-terminal, except for one GH19 chitinase (A0A5S3UPT5).

The genome of S4059 harbors nineteen predicted BGCs identified by antiSMASH 6.0, distributed with thirteen on chr I and six on chr II. BGC 2-5, 17, and 18 are non-ribosomal peptide synthetase clusters (NRPs), BGC 8, 13, and 16 are other unspecified ribosomally synthesized and post-translationally modified peptide products (RiPP) clusters, BGC 10, 11, and 15 are the hybrids of NRPs and Type I polyketide (PKs), BGC 7 is a hserlactone BGC, BGC 9 is prodigiosin BGC, and BGC 12, 13 belong to lanthipepride-class. Four of the BGCs located on chr I were predicted to produce indigoidine, kalimantacin A, amonabactin P 750, and prodigiosins (Table S1), however, only the prodigiosin gene cluster was predicted to be conserved to the known BGC with a similarity score of 70%, while the others were below 40% as shown in Table S1. The products of the rest of the BGCs are not known.

### 2.2. Global Proteome Profiles of P. rubra S4059 Grown on Chitin

**Figure 1:**
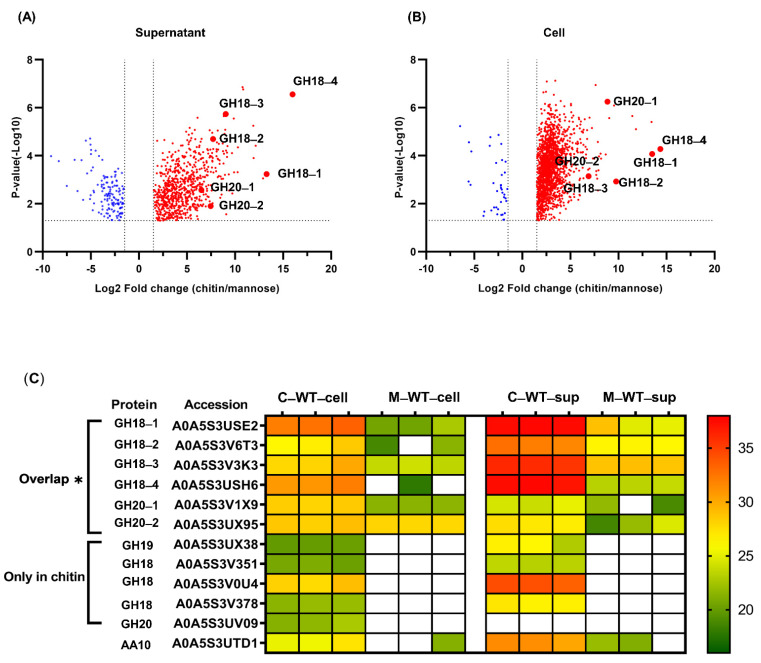
Comparison of identified proteins in the culture supernatant (**A**) and cells (**B**) of *Pseudoalteromonas rubra* S4059 when grown on chitin compared to on mannose. The dotted lines indicated that a threshold value for the cut off was a combination of *p*-value ≤ 0.05 and Log_2_ fold-change ≥ 1.5. Red dots represent upregulated proteins, and blue dots represent downregulated proteins. Chitinolytic enzymes with significant fold changes are highlighted by enlarged dots with protein names in (**A**,**B**). Heat map comparison of identified chitinolytic enzymes in the supernatant and cells of *Pseudoalteromonas rubra* S4059 is color-coded by increasing abundance (**C**). C–WT–cell: cells of S4059 grown on chitin; M–WT–cell: cells of S4059 grown on mannose; C–WT–sup: culture supernatant of S4059 grown on chitin; M–WT–sup: culture supernatant of S4059 grown on mannose. White denotes proteins not detected under this condition. ‘Overlap’ indicates these proteins were detected in both chitin and mannose-grown samples, and an asterisk (*) highlights proteins significantly upregulated when grown on chitin compared to on mannose.

The proteomes of *P. rubra* S4059 grown on different carbon sources were analyzed by liquid chromatography–tandem mass spectrometry (LC-MS/MS)-based label-free quantitative proteomics approach. The analyses were done on both cells and culture supernatant (excreted proteins) of S4059 grown in crystalline chitin or mannose containing medium. A total of 2813 proteins were identified in the global proteome of S4059, of which 738 and 142 proteins were up- and downregulated in S4059 culture supernatant when grown on crystalline chitin as compared to growth on mannose (Figure 1A). Simultaneously, 1861 and 141 proteins were up- and downregulated in S4059 cells when grown on crystalline chitin compared to on mannose (Figure 1B). Proteins involved in bacterial chemotaxis, cell division, flagellar assembly, and Type IV pilus assembly were upregulated when S4059 was grown on crystalline chitin (Table S3). Proteins associated with the core metabolism were also upregulated on chitin (Table S3). A protein involved in mannosidase from the GH92 family was upregulated when grown on mannose compared to when grown on crystalline chitin (Table S3).

### 2.3. Comparative Analysis of the Expression of Chitinolytic Enzymes in P. rubra S4059

A total of twelve chitin utilization associated proteins, including seven GH18s, three GH20s, a GH19, and a LPMO, were identified with a confidence rate of 99% at the peptide and protein levels in the *P. rubra* S4059 global proteome, while a GH19 (A0A5S3UPT5) and an LPMO (A0A5S3V4S2) were not detected (Figure 1C).

All chitinolytic enzymes could be detected in both cells and culture supernatant of S4059, except the enzyme A0A5S3UV09 from the GH20 family, which was not detected in the culture supernatant (Figure 1C). The GH18 chitinase A0A5S3USH6, annotated as ChiC, was the most highly expressed chitinolytic enzyme in both cells and culture supernatant during growth on chitin. Four GH18s (A0A5S3USE2, A0A5S3V6T3 A0A5S3V3K3, and A0A5S3USH6) and two GH20s (A0A5S3V1X9 and A0A5S3UX95) enzymes were significantly upregulated when S4059 was grown on crystalline chitin compared to on mannose (Figure 1). Meanwhile, several chitin-utilization associated proteins were induced and could only be detected when grown on crystalline chitin, including a GH19 chitinase and three GH18 chitinases (A0A5S3V351, A0A5S3V0U4, and A0A5S3V378) (Figure 1C). In addition, an LPMO from the AA10 family was identified under both growth conditions, but with no significant expression difference (Figure 1C).

### 2.4. Influence of Chitin on the Metabolome of S4059

To investigate the potential link between chitin degradation and secondary metabolite production, the S4059 wild type was grown on mannose, crystalline chitin, colloidal chitin, or N-acetyl glucosamine (NAG) containing marine minimal medium (MMM) in stationary phase. Chemical analysis using high-performance liquid chromatography coupled to diode array detection and high-resolution mass spectrometry (HPLC-DAD-HRMS) showed that mannose and crystalline chitin resulted in largely the same chemical profiles, although two unknown tentative prodigiosin analogs were produced in higher amounts on mannose (Figure S1). NAG containing media resulted in an overall higher production of secondary metabolites compared to mannose, crystalline, and colloidal chitin, and NAG and colloidal chitin both led to increased production of two unknown non-prodigiosin derived (based on UV-Vis absorption) compounds (Figure S1). Prodigiosin, hexyl prodigiosin, and heptyl prodigiosin (Figure S1B–D) were produced in varying amounts on all media. The identity of prodigiosin was confirmed using an authentic standard in combination with our in-house MS/MS library, and the two analogs were identified based on similarities with MS/MS and UV-Vis absorption spectra. Additionally, using our in-house MS/MS library, combined with a compound list generated from all *Pseudoalteromonas*-derived secondary metabolites found in the Reaxys database, no other known secondary metabolites were identified in S4059.

### 2.5. The Deletion of GH19 Chitinase Does Not Affect Growth or Chitin Degradation

**Figure 2:**
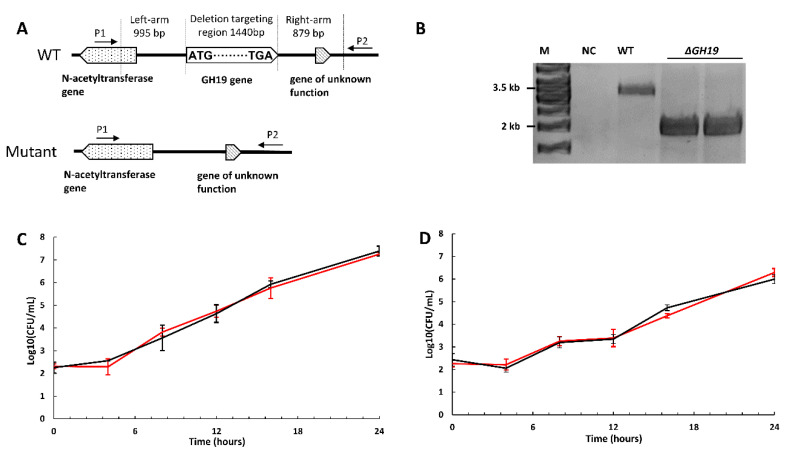
The in-frame deletion of the GH19 chitinase gene in *Pseudoalteromonas rubra* S4059 was verified by PCR with primers P1 and P2 that target the left and right homology arm of the GH19 chitinase gene (**A**). The PCR products were analyzed by electrophoresis (**B**). M: DNA ladder; NC: negative control with water as the PCR template; WT: PCR products with gDNA of wild type strain S4059 as a template; Δ*GH19*: PCR products with gDNA of Δ*GH19* as a template. Growth kinetics of *Pseudoalteromonas rubra* S4059 wild type (red lines) and Δ*GH19* (black lines) when grown in a marine minimal medium containing (**C**) colloidal chitin or (**D**) crystalline chitin (shrimp chitin) without casamino acids at 25 °C for 24 h, shaking at 200 rpm. Data were analyzed on three biological replicates, and the error bars represent the standard deviation.

To explore the function of GH19 chitinase in pigmented *P. rubra* S4059 and investigate a possible link between chitin degradation and secondary metabolites production, an in-frame deletion of GH19 chitinase gene (the GH19 A0A5S3UX38 with a signal peptide) was generated in S4059 by homologous recombination (Figure 2A,B). Wild type S4059 and GH19 deletion mutant (Δ*GH19*) were grown in MMM supplemented with crystalline chitin, colloidal chitin, NAG, or mannose. The mutant grew similarly to the wild type in all substrates with the same growth rate and maximum cell density (Figure 2C,D and Figure S2). The maximum cell density reached 10^9^ colony-forming unit (CFU)/mL in all substrates supplemented with casamino acid while only reaching 10^8^ CFU/mL when the strains grew in chitin containing MMM without casamino acid. The generation time of wild type and the mutant was 43.80 ± 8.46 min in all substrates with casamino acid, while without casamino acid, the value was 91.27 ± 2.23 min in crystalline chitin and 74.87 ± 0.30 min in colloidal chitin contained MMM.

The chitin degradation capacity of the *P. rubra* S4059 wild type and the GH19 mutant was also tested on chitin (crystalline and colloidal chitin) plates. As expected from the growth experiment, the mutant had the same chitinolytic ability (the size of clearing zone) as the wild type (Figure S2E–F). According to a previous study, the heterologously expressed GH19 chitinase in *E. coli* showed antifungal activity against *Aspergillus niger*. Therefore, the antifungal activity was also explored by co-cultivating both the strains with the fungus *Aspergillus niger* on marine agar (MA) plates, showing that the GH19 mutant displayed the same inhibitory effects as the wild type (data not shown).

### 2.6. Biofilm Formation and Chitin Surface Attachment of P. rubra S4059 Was Not Affected by Deletion of GH19 Chitinase Gene

Many *Pseudoalteromonas* species are good biofilm formers that are able to colonize crustaceans in marine environments [23], and since chitin colonization has been linked to biofilm formation in other bacteria [24], the biofilm formation of S4059 wild type and the mutant were compared in chitin containing media. The surface formed biofilm of the mutant and wild type was detected at the same level in all media (Figure S3).

**Figure 3:**
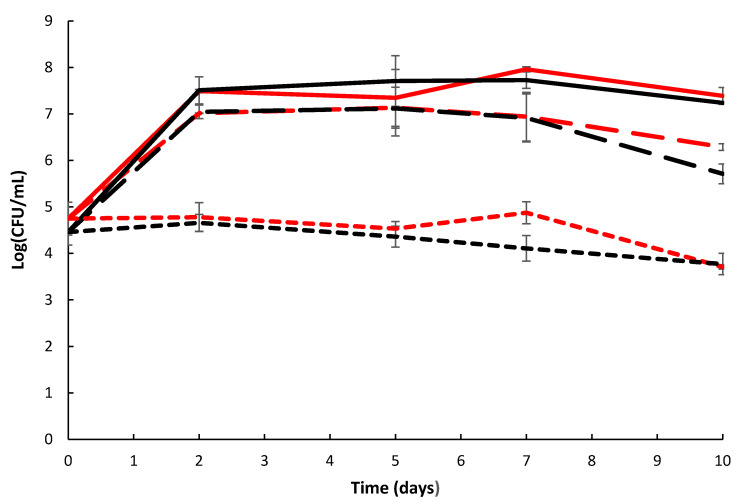
Growth and attachment of *Pseudoalteromonas rubra* S4059 wild type (red) and mutant ∆*GH19* (black) on Vannamei shrimp shells. Long dash lines: samples from liquid surrounding shrimp shells before sonication. Solid lines: samples from liquid surrounding shrimp shells after sonication. Short dash lines: samples from 3% Sigma sea salt without shrimp shells as a control. Data were analyzed on three biological replicates, and the error bars represent standard deviation.

The growth and attachment of the wild type and the mutant on natural chitin (shrimp shells) was assessed, and both strains grew from an initial density of 10^4^ CFU/mL to a final cell density of 10^7^ CFU/mL in the liquid surrounding the shells (Figure 3). Surface-attached bacteria were removed from shrimp shells by sonication resulting in an increase in cell density to 10^8^ CFU/mL with similar levels reached by wild type and the mutant (Figure 3).

### 2.7. Deletion of GH19 Chitinase Does Not Significantly Influence the Proteome of S4059

To investigate the impact of the GH19 chitinase deletion on the proteome of S4059, the GH19 mutant, and the wild type were grown on a chitin-based medium to stationary phase. Supernatant and cells were separated for proteome analysis. A total of 2512 proteins were identified, but no significant changes in any of the detected proteins were observed between wild type and GH19 mutant in either supernatant or cells when grown on crystalline chitin, except that GH19 chitinase could not be detected in the mutant cultures.

### 2.8. Similar Secondary Metabolome Pattern in Wild Type Strain and GH19 Mutant

Following the measurement of growth kinetics, the extracts of cultures grown in MMM were analyzed by HPLC-HRMS. Each strain was cultivated in three biological replicates in media containing one of the four carbon sources: mannose, NAG, colloidal chitin, or shrimp chitin. The wild type and the mutant had similar secondary metabolome profiles, as shown in Figure S1A. Screening for known natural products was also undertaken, revealing that only prodigiosin, as well as three of its analogs, could be identified (Figure S1B).

## 3. Discussion

Chitinolytic bacteria have been widely studied due to the possible use as antifungal agents in biocontrol [25]. In addition, the involvement of chitin in the ecology of *Vibrio cholerae* and its virulence regulation has been the focus of many studies [26,27,28]. Recently, a novel perspective on chitin degradation has been seen in some strains of *Vibrionaceae*, where growth on chitin as compared to on glucose leads to enhanced expression of several BGCs [19] along with enhanced antibacterial activity [20]. Chitin is the most abundant polymer in the ocean, and, hence, many marine bacteria are potent chitin degraders [10]. In silico analysis has demonstrated that pigmented species of the genus *Pseudoalteromonas* have elaborate chitinolytic machinery containing at least one of the otherwise rare GH19 chitinase in their genomes, and also, they devote up to 15% of their genome to BGCs with a vast potential for secondary metabolite production [7]. Here, we demonstrated that the pigmented bacterium *P. rubra* S4059 grew well using both purified chitin (crystalline and colloidal chitin) and natural shrimp shells as a sole source of nutrients. These results were further substantiated by proteome analyses that demonstrated that the strain S4059, indeed, produces an efficient chitinolytic enzyme cocktail, including GH18, GH19, LPMO, and GH20.

GH18 chitinases are the predominant chitinolytic enzymes in bacteria [29,30,31], and the strain S4059 harbored seven putative GH18 chitinases as determined by genome analysis, four of which were significantly upregulated when grown on chitin. Secretome analysis of the soil-derived and chitinolytic bacterium *Cellvibrio japonicus* indicated that many chitinolytic enzymes, including GH18 chitinases, and GH19 chitinases and LPMOs, were highly upregulated when grown on β-chitin as compared to α-chitin [29]. Out of fourteen putative chitinolytic enzymes in S4059, only the two putative enzymes, GH19 A0A5S3UPT5 and LPMO A0A5S3V4S2, were not detected when S4059 was grown on an α-chitin surface, suggesting that these two enzymes may have other functions. Proteome analysis of *P. rubra* S4059 culture supernatant confirmed the presence of all chitinolytic enzymes except for the two enzymes GH19 A0A5S3UPT5 and LPMO A0A5S3V4S2, and an even higher amount of the enzymes in culture supernatant than in cells (Figure 1C), implying that those detected proteins indeed are secreted enzymes able to degrade chitin. Secretome analysis of *Cellvibrio japonicus* showed that thousands of proteins were detected in the filtered culture supernatant. However, the authors mentioned that cell lysis can lead to overestimating the numbers of secreted proteins [29]. Further, they used the plate-based method that was developed by Bengtson et al. [32] to detect truly secreted proteins, and the result indicated that only 267 secreted proteins were detected in α-chitin containing medium [29]. In our study, due to the insoluble property of chitin, secreted chitinases may bind to small chitin particles, and, thus, the secreted chitinases cannot be passed through sterile filters leading to misestimating the expression of secreted chitinases. Thus, we did not filter the culture supernatant for retaining bound chitinases, which were on chitin particles. We found that 880 proteins were influenced by chitin in the culture supernatant of S4059, as compared to on mannose, and these high numbers of influenced proteins may be due to a combination factor of the unfiltered culture supernatant and cell lysis during centrifugation [33]. Further, the agar plate-based method probably can point out the true numbers of secreted proteins in S4059.

One of the two GH19 chitinases in *P. rubra* S4059 was highly expressed when the bacterium was grown on chitin, and while some GH19 chitinases have been linked to antifungal activity, their broader role in bacteria remains enigmatic [18,34,35]. The results suggesting antifungal activity have mainly relied on heterologous expression of GH19 in hosts such as *E. coli,* and this provides information about the enzyme per se, but not necessarily about the actual role in its native host. We, therefore, chose to delete the GH19 that was highly expressed on chitin to explore its possible role in S4059 further. Both the mutant and wild type displayed similar antifungal activity (data not shown). Despite the high expression of GH19 when S4059 was grown on chitin, the GH19 deficient mutant showed no difference in growth compared to the WT when grown on chitin (Figure 2 and Figure S2). Our results, therefore, indicate that GH19 chitinase is not the predominant chitin degrading enzyme in *P. rubra* S4059, given the wide array of other chitinolytic enzymes. Chitin utilization capacity has been investigated in a chitinolytic soil bacterium *Cellvibrio japonicus* [30]. Through a combination of secretome and genetic manipulation approaches, a highly expressed GH18 chitinase was identified as the enzyme essential for the degradation of chitin in the strain [30]. Proteome analyses in S4059 demonstrated that two GH18 chitinases A0A5S3USH6 and A0A5S3USE2 are the most highly expressed chitinases in both cells and culture supernatant according to protein abundance when S4059 was grown on chitin (Table S7), suggesting that the GH18 chitinases could be potentially essential enzymes for chitin degradation in S4059 under testing conditions.

In conclusion, the proteomic analysis showed that a highly efficient chitin degradation machinery was identified in pigmented *P. rubra* S4059, and four GH18 chitinases and two GH20 hexosaminidases in S4059 were significantly upregulated when grown on chitin. In contrast to different proteome profiles, the different growth conditions on chitin investigated here did not significantly alter the metabolite profile of S4059, in contrast to what has been reported in other *Vibrio* species and *Streptomyces coelicolor* A3 (2) [19,20,21,22]. Although the deletion of GH19 chitinase did not influence chitin degradation activity in S4059, we developed a conjugation-based approach allowing genetic manipulation in this bioactive bacterium S4059. Given the large potential for secondary metabolism as found by genome mining and from uncharacterized compounds produced on especially chitin derived substrates, the genetic approach developed here will allow exploration of the novel chemical space of this organism.

## 4. Materials and Methods

### 4.1. Bacterial Strains, Plasmids, and Growth Conditions

All bacterial strains and plasmids used in this study are listed in Table S5. *P. rubra* S4059 was isolated during the Galathea 3 expedition [36] and cultured in marine broth (MB, BD Difco 2216, Le Pont de Claix, France) or APY medium [37] at 25 °C, 200 rpm. *P. rubra* S4059 carrying a chromosomal-integrated suicide plasmid was cultured in MB containing 30 µg/mL chloramphenicol (Sigma, C0378, St. Louis, MO, USA) at 25 °C, 200 rpm. All chitin used in this study are α-chitin. *P. rubra* S4059 mutants and wild type were cultured in a marine minimal medium (MMM) [38] supplemented with four different carbon sources (0.2% mannose, 0.2% crystalline chitin, 0.2% colloidal chitin, or 0.2% NAG) and growth, biofilm formation, and secondary metabolome determined. To compare chitin degradation of mutants and wild type S4059, they were grown on plates containing 2% Sea Salt (Sigma, S9883, St. Louis, MO, USA), 1.5% agar, and 0.2% chitin (crystalline or colloidal). Colloidal chitin was prepared as described in a previous study [39].

All *Escherichia coli* strains were cultured in Luria Bertani (LB) Broth (BD Difco 244520, Le Pont de Claix, France) at 37 °C, 200 rpm. *E. coli* GB *dir*-*pir*116 [40] was used for constructing suicide plasmid. *E. coli* WM3064 was used as the donor strain in intergeneric conjugation experiments. *E. coli* WM3064 is a *dapA* mutant that requires exogenously supplied diaminopimelic acid (DAP, Sigma, D1377, St. Louis, MO, USA) with a final concentration of 0.3 µM for growth [41]. Plasmid pDM4 was used as the backbone of suicide vectors [42]. *E. coli* strains harboring pDM4, or its derivatives were grown in LB Broth with 10 µg/mL chloramphenicol or in LB agar with 15 µg/mL chloramphenicol.

### 4.2. Whole Genome Sequencing and Assembly of P. rubra S4059

Genomic DNA of *P. rubra* S4059 was extracted using the Genomic DNA buffer set (QIAGEN, 19060, Hilden, Germany) following the supplier’s instructions. The closed genome of *P. rubra* S4059 was obtained by minION sequencing using the EXP-FLP002 flow cell priming kit, SQK-RAD004 rapid sequencing kit, FLO-MINSP6 flow cell, and the associated protocol (version RSE_9046_V1_revB-17Nov2017 and RSE_9046_V1_revB-14 AUG2019). lllumina reads, obtained from a previous study [7], were combined with the minION reads for hybrid assembly using the Unicycler package [43]. Before assembly, the minION reads were filtered using the Filtlong package, only keeping the top 500,000,000 bp. The genome was annotated using Prokka [44]. The genome is available at the National Center for Biotechnology Information (NCBI) under the accession number CP045429 and CP045430.

### 4.3. In Silico Analysis of Chitin Degrading Genes and Secondary Metabolites

The assembled genome was analyzed using the CLC Main Workbench 8.0.1 (CLC bio, Aarhus, Denmark) and the online platform MaGe MicroScope [45]. The chitinolytic enzymes and the prediction of the chitin degradation pathway in S4059 were annotated according to the prediction of the S4059 proteome in Uniprot (UniProt ID UP000305729). Amino acid sequences of the candidate chitinases were also submitted to the SignalP 5.0 Server [46] to identify the signal peptides. In addition, genomes were submitted to antiSMASH version 6.0 (https://antismash.secondarymetabolites.org/#!/start (accessed on 15 January 2021)) for the prediction of putative biosynthetic gene clusters involved in the production of secondary metabolites.

### 4.4. Growing Bacteria and Sample Preparation for Proteomic Analyses

The protein samples were prepared according to Chevallier et al. [47]. *P. rubra* S4059 wild type and GH19 mutant were grown in 20 mL MMM with crystalline chitin for 2 days at 25 °C, 200 rpm. All experiments were carried out in biological triplicates. A five milliliter culture was harvested (5000× *g*, 20 min), and then the culture supernatant was transferred into a new 15 mL Falcon tube, and ice-cold acetone (−20 °C) was added to the supernatant to a final concentration of 80%. Then the mixture was kept at −20 °C overnight and harvested at 2000× *g* for 20 min. Acetone was carefully removed. The harvested bacterial cells were washed twice with ice-cold phosphate-buffered saline (PBS), and the pellet was lysed using 20 µL of lysis buffer (consisting of 6 M Guanidinium Hydrochloride, 10 mM Tris (2-carboxyethyl) phosphine hydrochloride, 40 mM 2-chloroacetamide, 50 mM HEPES (4-(2-hydroxyethyl)-1-piperazineethanesulfonic acid) pH 8.5). Samples were inactivated at 95 °C for 5 min and were then sonicated on high 3 times for 10 s in a 4 °C Bioruptor sonication water bath (Diagenode). A Bradford assay (Sigma) was used to determine protein concentration, and 50 µg of each sample was used for digestion. Samples were diluted 1:3 with 10% Acetonitrile, 50 mM HEPES pH 8.5, LysC (MS grade, Wako, Japan) added in a 1:50 (enzyme to protein) ratio, and samples were incubated at 37 °C for 4 h. Samples were further diluted to 1:10 with 10% Acetonitrile, 50 mM HEPES pH 8.5, trypsin (MS grade, Promega) added in a 1:100 (enzyme to protein) ratio, and samples were incubated overnight at 37 °C. Enzyme activity was quenched by adding 2% trifluoroacetic acid (TFA) to a final concentration of 1%. Before mass spectrometry analysis, the peptides were desalted on SOLAu C18 plates (ThermoFisher Scientific, Roskilde, Denmark). After each solvent application, the plate was centrifuged for 1 min at 350× *g*. For each sample, the C18 material was activated with 200 µL of 100% Methanol (HPLC grade, Sigma), then 200 µL of 80% Acetonitrile, 0.1% formic acid. The C18 material was subsequently equilibrated 2 x with 200 µL of 1% TFA, 3% Acetonitrile, after which the samples were loaded. After washing the tips twice with 200 µL of 0.1% formic acid, the peptides were eluted using 40% Acetonitrile, 0.1% formic acid, and transferred into clean 500 µL Eppendorf tubes. The eluted peptides were concentrated in an Eppendorf Speedvac and reconstituted in 1% TFA, 2% Acetonitrile for Mass Spectrometry (MS, Merck, Darmstadt, Germany) analysis.

### 4.5. Proteomic Data Acquisition

The proteomic data acquisition approach was modified from Haddad Momeni et al. [48]. Briefly, peptides were loaded onto a 2 cm C18 trap column (ThermoFisher 164705), connected in-line to a 15 cm C18 reverse-phase analytical column (Thermo EasySpray ES803) using 100% Buffer A (0.1% Formic acid in water) at 750 bar, using the Thermo EasyLC 1200 HPLC system (ThermoFisher, Roskilde, Denmark), and the column oven operating at 30 °C. Peptides were eluted over a 140 min gradient ranging from 10 to 60% of 80% acetonitrile, 0.1% formic acid at 250 nL/min, and a Q-Exactive instrument (ThermoFisher Scientific, Roskilde, Denmark) was run in a DD-MS2 top 10 method. Full MS spectra were collected at a resolution of 70,000, with an automatic gain control (AGC) target of 3 × 10^6^ or maximum injection time of 20 ms and a scan range of 300 to 1750 *m*/*z*. The MS2 spectra were obtained at a resolution of 17,500, with an AGC target value of 1 × 10^6^ or maximum injection time of 60 ms, a normalized collision energy of 25, and an intensity threshold of 1.7 × 10^4^. Dynamic exclusion was set to 60 s, and ions with a charge state <2 or unknown were excluded. MS performance was verified for consistency by running complex cell lysate quality control standards, and chromatography was monitored to check for reproducibility. The mass spectrometry data have been deposited to the ProteomeXchange Consortium (http://proteomecentral.proteomexchange.org (accessed on 21 December 2020)) via the PRIDE partner repository with the dataset identifier PXD 023249. The reviewer account details: Username: reviewer_pxd023249@ebi.ac.uk; Password: xteOmcDd. The raw files were analyzed using Proteome Discoverer 2.4. Label-free quantitation (LFQ) was enabled in the processing and consensus steps, and spectra were matched against the *P. rubra* S4059 database obtained from Uniprot (UniProt ID: UP000305729). Dynamic modifications were set as Oxidation (M), Deamidation (N, Q), and Acetyl on the protein N-termini. Cysteine carbamidomethyl was set as a static modification.

The methods of proteomic analyses were modified from Beyene et al. [49]. Briefly, all the statistical analyses were performed using Perseus software (version 1.6.14.0, MaxPlanck Institute of Biochemistry, Martinsried, Germany), https://maxquant.net/perseus/ (accessed on 20 September 2020)), and all results were filtered to a 1% false discovery rate (FDR). The normalized abundance values for each protein were log_2_ transformed, and at least two valid values were required in the bio-triplicates for quantitation. When the original signals were zero, they were imputed with random numbers from a normal distribution, in which the mean and standard deviation were chosen from low abundance values below the noise level (Width = 0.3; shift = 1.8) [49,50]. To identify proteins with significantly different abundances when S4059 were grown on chitin compared to on mannose, the FDR were estimated using Benjamini–Hochberg method, and a two-tailed unpaired *t*-test was used with a combination of *p*-value ≤ 0.05 and Log_2_ fold-change ≥1.5 [51]. The resulting significant proteins were exported from Perseus and visualized in GraphPad Prism 8 (Graphpad Software, San Diego, CA, USA, https://www.graphpad.com/scientific-software/prism/ (accessed 5 October 2020)) using volcano plots.

### 4.6. DNA Manipulation

Genomic DNA of *P. rubra* S4059 was extracted using the Genomic DNA buffer set (QIAGEN, 19060, Hilden, Germany), as mentioned above. All primers used in this study are listed in Table S6. All purified DNA fragments were amplified using PrimeSTAR^®^ Max Premix (TaKaRa, catalog number: R045A, Kusatsu, Japan). Blue TEMPase Hot Start Master Mix K (catalog number: 733-2584, Haasrode, Belgium) was used for homologous recombination event checking by PCR. All primers and plasmids were designed in A Plasmid Editor-ApE. The specificity of primers was checked by BLAST against the *P. rubra* S4059 genome. All primers were ordered from Integrated DNA technologies (Leuven, Belgium).

### 4.7. Construction of Suicide Plasmids for in-Frame Deletion of GH19 Chitinase in P. rubra S4059

The suicide plasmid was constructed by the direct cloning method using pDM4 as the backbone [40,42]. The pDM4 plasmid contains an R6K replicon origin, *mob* genes and *oir*T for conjugation, and a chloramphenicol resistance gene *cat* and a *sacB* gene for counter selection [42]. An approximately 1-kb upstream and downstream region flanking of GH19 gene was amplified with primer pairs GH19-L-F/GH19-L-R, GH19-R-F/GH19-R-R (Table S6). The amplified recombining arms were fused with overlap extension PCR to form the recombining arm segments, which were cloned into pJET1.2 subcloning vector using a CloneJET PCR Cloning Kit (ThermoFisher Scientific, K1231, Vilnius, Lithuania) for sequencing. Subsequently, the homologous segment was amplified from sequencing-confirmed pJET1.2-dGH19 arms using primers GH19-pJET1.2-F and GH19-pJET1.2-R. The linear backbone was amplified from the pDM4 suicide vector using primers (GH19-pDM4-F/GH19-pDM4-R). After gel purification, the linear vector and the homologous fragment were co-electroporated into *E. coli* GB *dir-pir116* and ligated by the RecET direct cloning system [40]. The restriction cloning method was also attempted several times to construct this plasmid. However, it was unsuccessful. All plasmids were extracted using a QIAprep Spin Miniprep Kit (QIAGEN, 27106, Hilden, Germany).

### 4.8. Conjugation of P. rubra S4059

The conjugation protocol was modified from Yu et al. and Wang et al. [52,53]. *E. coli* WM3064 harboring the suicide plasmid were used as the donor and *P. rubra* S4059 as the recipient. Overnight cultures of donor and recipient were prepared as pre-cultures one day before the conjugation. During conjugation, both strains were diluted 100 times and grown to OD_600_ ≈ 0.6. One-mL donor cells were harvested at 6000× *g* for 1 min. The pellets were resuspended and washed once using 1 mL LB + DAP. Then, 1 mL recipient was added to the *E. coli* WM3064 pellet and centrifuged at 6000× *g* for 1 min. One-mL MB + DAP was added to wash the mixture by pipetting and centrifuging at 6000× *g* for 1 min. The supernatant was removed until 20–30 µL liquid remained. The mixture of cells was resuspended and placed on a 0.2-μm pore-size membrane (MF-Millipore, GSWP02500) that was placed on an APY + DAP agar plate. The mating plates were incubated at 20 °C for 24 h. The cells were suspended in 1 mL MB and incubated with shaking at 750 rpm in an Eppendorf^®^ thermomixer comfort, 25 °C for 1 h. After recovery, cells were spread on MA plates with 30 μg/mL chloramphinical (Table S4) and incubated at 25 °C for 24–48 h.

### 4.9. Confirmation of the First Crossing over Mutants and Deletion Mutants

Colonies from the first crossover selective plates were picked and cultured in 5 mL MB containing 30 µg/mL chloramphenicol overnight. Genomic DNA extraction from pre-cultures using NucleoSpin^®^ Tissue kit (Macherey–Nagel, Düren, Germany, 740952.250). To determine whether the plasmid integrated into target regions, the genomic DNA was used as the template for PCR checking with primers (Cm^r^-F/Cm^r^-R, GH19-p 1/GH19-p 4, and GH19-p 2/GH19-p 3). Colonies carrying the integrated plasmid were cultured in MB with 30 µg/mL chloramphenicol at 25 °C overnight as pre-culture. The pre-culture was diluted 100 times and inoculated in 5 mL fresh MB without antibiotics until OD_600_ ≈ 0.6. The culture was 10-fold diluted and spread on the counter selection plates (half nutrients of MA) containing 10% sucrose. These counter selection plates were incubated at 20 °C until colonies were visible. Confirmation of the in-frame deletion mutants was carried out by PCR application and sequencing. Primers (GH19-p1/GH19-p2) were designed to amplify the mutation region, and the PCR produced was purified and sent for DNA sequencing.

### 4.10. Growth Curves of Wild Type and Mutant

Growth kinetics of *P. rubra* S4059 WT (wild type) and Δ*GH19* (GH19 chitinase mutant) were established in MMM with or without 0.3% casamino acids and supplemented with 0.2% colloidal chitin, 0.2% crab chitin (C9752, Sigma, St. Louis, MO, USA), 0.2% mannose (63580, Sigma, St. Louis, MO, USA), or 0.2% NAG (A3286, Sigma, St. Louis, MO, USA), respectively, as carbon source. Pre-cultures of *P. rubra* WT and mutants were grown in MB overnight at 25 °C. The cultures were diluted to a starting concentration of 10^3^ CFU/mL in MMM. Samples were taken every 4 h for estimation of cell density by plate counting. All experiments were done in three biological replicates.

### 4.11. Growth and Attachment on Shrimp Shells

Six millimeter diameter circular disks were prepared from the exoskeleton of Vannamei shrimps (description in Supplementary Material). Bacterial cultures (WT and mutants) were grown in 5 mL MB in 50 mL flasks at 25 °C, 200 rpm overnight. The cultures were diluted to 10^4^ CFU/mL in artificial seawater (3% sea salt, Sigma, S9883) and incubated with or without shrimp shells in 1.5 mL Eppendorf tubes. Samples of the suspension were taken after 0, 2, 5, 7, 10 days incubation and plated after serial dilution. The tubes were sonicated five minutes at 50/60 Hz in an ultrasonic bath (Emmi D20 Q, EMAG, Mörfelden-Walldorf, Germany) and vortexed 10 s to remove bacteria attached to the shrimp shells [54]. A suspension of cells with known cell counts was subjected to the same sonication and cell counts done after sonication, demonstrating that this procedure did not reduce cell counts. Then serial dilutions and colony counts were done to determine cell densities. The experiment was done in biological duplicate.

### 4.12. Extraction of Metabolites for Chemical Analysis

Bacterial cultures were grown in MMM with different carbon sources, and samples were taken for chemical analyses after 72 h incubation. Ten milliliters of the sample were extracted with an equal volume of high-performance liquid chromatography (HPLC)-grade Ethyl acetate in 50 mL Falcon tubes. The organic phase was transferred to a new vial and evaporated under nitrogen. The dried extract was re-dissolved in 500 μL methanol and stored at −20 °C. Chemical analysis was performed in biological triplicate.

### 4.13. Chemical Analysis by UHPLC-HRMS

The chemical analysis approach was modified from Holm et al. [55]. Chemical analysis was performed on a Bruker maXis 3G orthogonal acceleration quadrupole time-of-flight mass spectrometer (Bruker Daltonics, Billlerica, MA, USA) equipped with an electrospray ionization (ESI) source and connected to an Ultimate 3000 UHPLC system (Dionex, Sunnyvale, CA, USA). The column used was a reverse-phase Kinetex 1.7 μm C18, 100 mm × 2.1 mm (Phenomenex). The column temperature was kept at 40 °C throughout the analysis. A linear gradient of LC-MS grade water and acetonitrile both buffered with formic acid was used, starting at 10% (*v/v*) acetonitrile and increased to 100% in 10 min, maintaining this rate for 3 min before returning to the starting conditions in 0.1 min and staying there for 2.4 min before the following run. A flow rate of 0.40 mL/min was used. Time-of-flight mass spectrometry (TOFMS) was performed in ESI+ with a data acquisition range of 10 scans per second at *m*/*z* 75–1250. The TOFMS was calibrated using the Bruker Daltonics high precision calibration algorithm by means of the internal standard sodium formate, which was automatically infused before each run. This provided a mass accuracy of better than 1.5 ppm in MS mode. UV-visible spectra were collected at wavelengths from 200 to 700 nm. Data processing was performed using DataAnalysis 4.0 (Bruker Daltonics, Billerica, MA, USA) and Target Analysis 1.2 software (Bruker Daltonics). Tandem MS spectra were acquired on an Agilent 6545 QTOF-MS using the method described in Isbrandt et al. (2020) [56].

## Figures and Tables

**Table 1:** The chitinolytic machinery in *Pseudoalteromonas rubra* S4059 according to the prediction of the proteome from Uniprot (Proteome ID UP000305729). The signal peptide was predicted using amino acid sequence by SignalIP-5.0 (http://www.cbs.dtu.dk/services/SignalP/ (accessed on 22 September 2018)).

Glycoside Hydrolase Type	Accession	Signal Peptide
GH18	A0A5S3USE2	Y
	A0A5S3V351	Y
	A0A5S3V6T3	Y
	A0A5S3V0U4	Y
	A0A5S3USH6	Y
	A0A5S3V3K3	Y
	A0A5S3V378	Y
GH19	A0A5S3UX38	Y
	A0A5S3UPT5	N
GH20	A0A5S3UX95	Y
	A0A5S3UV09	Y
	A0A5S3V1X9	Y
LPMO	A0A5S3UTD1	Y
	A0A5S3V4S2	Y

## Data Availability

Proteomic data are available via ProteomeXchange with identifier PXD023249 (http://www.proteomexchange.org/) and the genome of *Pseudoalteromonas rubra* S4059 is available at the National Center for Biotechnology Information (NCBI) under the accession number CP045429 (https://www.ncbi.nlm.nih.gov/nuccore/CP045429.1/ (accessed on 23 November 2020)) and CP045430 (https://www.ncbi.nlm.nih.gov/nuccore/CP045430.1/ (accessed on 23 November 2020)). Further inquiries can be directed to the corresponding author/s.

## Supplementary Materials

The following are available online at https://www.mdpi.com/1660-3397/19/2/108/s1, Table S1. The predicted biosynthetic gene clusters (BGCs) of Pseudoalteromonas rubra S4059 by antiSMASH 6.0. Table S2. The fold change of six significantly upregulated chitinolytic enzymes in cell or supernatant samples. Table S3. Significantly up-and downregulated proteins in Pseudoalteromonas rubra S4059 proteome when grown on chitin compared to on mannose. Table S4. Antibiotic sensitivity of Pseudoalteromonas rubra S4059 growth on an MB agar plate. Table S5. Bacteria and plasmids used in this study. Table S6. Primers used in this study. Table S7. The normalized protein abundance of chitinolytic enzymes in S4059. Figure S1. (A) Base peak chromatograms of Pseudoalteromonas rubra S4059 wild type (WT) and ΔGH19 mutant when cultivated on a marine minimal medium using mannose, crystalline chitin, colloidal chitin, or N-acetyl glucosamine (NAG) as the carbon source. The red pigment prodigiosin and two of its analogs (hexyl prodigiosin and heptyl prodigiosin) could be identified in the extract and confirmed based on MS/MS experiments and the acquired absorption spectra. Experiments were done in biological triplicates, and a reference chromatogram of the sterile growth medium was included to show media components also present in the experiments. (B) Tandem MS spectra recorded for (1) prodigiosin, (2) hexyl-prodigiosin, and (3) heptyl-prodigiosin. The prodigiosin MS/MS spectra matched our in-house MS/MS library, and the characteristic fragment m/z 252.11 and loss of CH3 (m/z 15.02) additionally matched those previously reported for prodigiosin and analogs [57]. (C) Recorded UV-Vis absorption spectrum of prodigiosin. All proposed prodigiosins share identical absorption spectra. The spectrum is in agreement with previously reported literature [58]. Figure S2. (A–D) Growth kinetics of Pseudoalteromonas rubra S4059 wild type (WT) and ΔGH19 mutant in mannose (A), colloidal chitin (B), crystalline chitin (crab chitin) (C), and NAG (chitin monomer) (D) containing a marine minimal medium (MMM) with casamino acids. (E,F) WT and ΔGH19 growth in crystalline chitin (E) or colloidal chitin containing MMM without casamino acid. Square: WT; Triangle: ΔGH19. The points are bio-replicates, and error bars are standard deviation. (G,H) Chitin degradation activities of wild type and the mutant on colloidal chitin plate (G) and crystalline chitin plate (H). Figure S3. Biofilm formation on the microtiter-well plastic surface of Pseudoalteromonas rubra S4059 wild type and ΔGH19 mutant in four different sole carbon contained medium as determined by the O’Toole and Kolter crystal violet assay. (A) in mannose; (B) in NAG (chitin monomer); (C) in colloidal chitin; (D) in crab chitin. All experiments were in bio-triplicates, and error bars are standard deviation.
