# Supplementary material for: Chitin Degradation Machinery and Secondary Metabolite Profiles in the Marine Bacterium Pseudoalteromonas rubra S4059
Source: Mar Drugs. 2021 Feb 12;19(2):108. doi: 10.3390/md19020108 (PMC7917724; doi:10.3390/md19020108)
Supplement: Supplementary file 1 [file marinedrugs-19-00108-s001.pdf]

Supplementary Materials

Chitin degradation machinery and secondary metabolite profiles in the marine bacterium *Pseudoalteromonas rubra* S4059

Xiyan Wang [1], Thomas Isbrandt [1], Mikael Lenz Strube [1], Sara Skøtt Paulsen [1], Maike Wennekers Nielsen [1], Yannick Buijs [1], Erwin M. Schoof [1], Thomas Ostenfeld Larsen [1], Lone Gram [1] and Shengda Zhang [1] *

[1] Department of Bioengineering, Technical University of Denmark, DK-2800 Kgs. Lyngby, Denmark; xwan@dtu.dk (X.W.); tispe@bio.dtu.dk (T.I.); milst@dtu.dk (M.L.S.); saskp@dtu.dk (S.S.P.); mweni@dtu.dk (M.W.N.); erws@dtu.dk (E.M.S.); yabu@dtu.dk (Y.B.); tol@bio.dtu.dk (T.O.L); gram@bio.dtu.dk (L.G.).

* Correspondence: shez@dtu.dk (S.Z.); Tel.: +45-5011-7765.

* Corresponding author:

Sheng-Da Zhang

Department of Biotechnology and Bioengineering, Technical University of Denmark, Søltofts Plads bldg. 221, DK-2800 Kgs. Lyngby, Denmark

E-mail: shez@dtu.dk Telephone: +45-50117765

Supplementary Results

Anti-fungal activity

P. rubra S4059 WT and Δ*GH19* were tested against *Aspergillus niger* in an antifungal assay. Both strains were antifungal against *A. niger*. However, there is no signficant difference between WT and *ΔGH19* and neither of them could retain the antifungal activity over time.

Table and Figure legends

Table S1. The predicted biosynthetic gene clusters (BGCs) of *Pseudoalteromonas rubra* S4059 by antiSMASH 6.0.

Table S2. The fold change of six significantly upregulated chitinolytic enzymes in cell or supernatant samples.

Table S3. Significantly up-and down-regulated proteins in *Pseudoalteromonas rubra* S4059 proteome when grown on chitin as compared to on mannose.

Table S4. Antibiotic sensitivity of *Pseudoalteromonas rubra* S4059 growth on MB agar plate.

Table S5. Bacteria and plasmids used in this study

Table S6. Primers used in this study.

Table S7. The normalized protein abundance of chitinolytic enzymes in S4059.

Figure S1. (A) Base peak chromatograms of *Pseudoalteromonas rubra* S4059 wild type (WT) and *ΔGH19* mutant when cultivated on marine minimal medium using mannose, crystalline chitin, colloidal chitin or N-acetyl glucosamine (NAG) as the carbon source. The red pigment prodigiosin and two of its analogues (hexyl prodigiosin and heptyl prodigiosin) could be identified in the extract and confirmed based on MS/MS experiments and the acquired absorption spectra. Experiments were done in biological triplicates, and a reference chromatogram of the sterile growth medium is included to show media components also present in the experiments. (B) Tandem MS spectra recorded for 1) prodigiosin, 2) hexyl-prodigiosin, and 3) heptyl-prodigiosin. The prodigiosin MS/MS spectra matches our in-house MS/MS library, and the characteristic fragment m/z 252.11, and loss of CH_3 (m/z 15.02) additionally matches those previously reported for prodigiosin and analogues [5]. (C) Recorded UV-Vis absorption spectrum of prodigiosin. All proposed prodigiosins share identical absorption spectra. The spetrum is in agreement with previously reported literature [6]

Figure S2. (A-D) Growth kinetics of *Pseudoalteromonas rubra* S4059 wild type (WT) and *ΔGH19* mutant in mannose (A), colloidal chitin (B), crystalline chitin (crab chitin) (C) and NAG (chitin monomer) (D) containing MMM with casamino acids. (E-F) WT and *ΔGH19* growth in crystalline chitin (E) or colloidal chitin containing MMM without casamino acid. Square: WT; Triangle: *ΔGH19*. The points are bio-replicates and error bars are standard deviation. (G-H) Chitin degradation activities of wild type and the mutant on colloidal chitin plate (G) and crystalline chitin plate (H).

Figure S3. Biofilm formation on the microtiter-well plastic surface of *Pseudoalteromonas rubra* S4059 wild type and Δ*GH19* mutant in four different sole carbon contained medium as determined by the O'Toole & Kolter crystal violet assay. (A) in mannose; (B) in NAG (chitin monomer); (C) in colloidal chitin; (D) in crab chitin. Each experiments are repeated in bio-triplicates and error bars are standard deviation.

Table S1. The predicted biosynthetic gene clusters (BGCs) of *Pseudoalteromonas rubra* S4059 by antiSMASH 6.0.

	Type	From	To	Most similar known cluster		Similarity
**chrI**						
BGC1	RRE-containing	484,852	504,385			
BGC2	NRPS-like	665,623	707,167			
BGC3	NRPS	1,025,623	1,124,309			
BGC4	NRPS-like	1,152,132	1,194,273	indigoidine	NRP	40%
BGC5	NRPS	1,204,311	1,261,426			
BGC6	T3PKS	1,684,665	1,725,708			
BGC7	hserlactone	2,486,553	2,507,302			
BGC8	RiPP-like	2,814,767	2,824,604			
BGC9	prodigiosin	3,203,980	3,239,003	prodigiosin		70%
BGC10	NRPS,T1PKS,transAT-PKS	3,627,019	3,764,918	kalimantacin A	NRP + Polyketide: Modular type I + Polyketide: Trans-AT type I	10%
BGC11	NRPS,T1PKS,betalactone,thioamide-NRP	4,235,481	4,358,990	amonabactin P 750	NRP	42%
BGC12	lanthipeptide-class-i	4,533,397	4,557,768			
BGC13	RiPP-like	4,561,329	4,572,171			
**chrII**						
BGC14	lanthipeptide-class-iv	165,032	187,755			
BGC15	NRPS,T1PKS	209,321	279,019			
BGC16	RiPP-like	629,147	639,983			
BGC17	NRPS	864,063	1,000,602			
BGC18	RRE-containing	1,025,566	1,047,998			
BGC19	NRPS	1,052,424	1,095,471			

Table S2. The fold change of six significantly upregulated chitinolytic enzymes in cell or supernatant samples.

	Glycoside hydrolase type	Log_2(Fold change)
cells samples	GH18-1	13.5
	GH18-2	9.76
	GH18-3	6.89
	GH18-4	14.36
	GH20-1	8.86
	GH20-2	2.74
supernatant samples	GH18-1	13.27
	GH18-2	7.71
	GH18-3	9.02
	GH18-4	15.97
	GH20-1	6.49
	GH20-2	7.46

Table S3. Significantly up-and down-regulated proteins in *Pseudoalteromonas rubra* S4059 proteome when grown on chitin as compared to on mannose.

Function	Log2(Fold change)	Accession	Protein
Pilus assembly	+2.80	A0A5S3V0R4	Pilin biogenesis protein
	+1.97	A0A0U2P9L9	Pilus assembly protein PilM
	+2.68	A0A5S3UZ53	Pilus assembly protein PilN
	+1.65	A0A0L0EQN7	Pilus assembly protein PilP
	+2.65	A0A5S3UZ07	Pilus assembly protein PilP
	+2.47	A0A5S3V1T9	Pilus assembly protein PilW
	+3.10	A0A5S3V0T8	Pilus assembly protein PilX
	+2.62	A0A5S3UQS3	Pilus assembly protein PilZ
	+2.01	A0A5S3UWH8	PilZ domain-containing protein
	+1.86	A0A5S3URP5	PilZ domain-containing protein
	+5.68	A0A5S3URS7	PilZ domain-containing protein
Chemotaxis and Flagellar assembly	+3.09	A0A0L0EQP9	Chemotaxis protein CheX
	+4.43	A0A0U3HPG4	Flagellar motor switch protein FliG
	+2.82	A0A0L0ERK8	Flagellar motor switch protein FliN
	+4.19	A0A0L0EU84	Chemotaxis protein CheY
	+3.27	A0A0F4QWI4	Chemotaxis protein CheY
	+2.80	A0A0L0EV12	Chemotaxis protein CheY
	+3.25	A0A0U3I1F8	Chemotaxis protein CheV
	+2.73	A0A0L0EW69	Fis family transcriptional regulator
	+1.81	A0A0F4QWB6	Chemotaxis protein CheW
	+1.26	A0A0F4QWN8	Flagellar basal-body rod protein FlgG
	+4.90	A0A0L0ERQ9	Flagellar motor switch protein FliM
	+4.17	A0A5S3UYM4	Flagellar assembly protein FliH
	+3.85	A0A5S3UTL8	Flagellar biogenesis protein
	+3.62	A0A5S3UXB9	Flagellar M-ring protein
	+3.52	A0A5S3UX71	Flagellar protein FliL
	+2.84	A0A5S3UQH6	Flagellar motor protein MotA
	+2.06	A0A5S3UTK5	Flagellar biosynthesis protein FlgP
	+2.05	A0A0L0EPQ6	Flagellar motor protein PomA
	+1.89	A0A5S3UX07	Flagellar hook protein FlgE
	+1.66	A0A5S3UWZ7	Flagellar L-ring protein FlgH
	+1.48	A0A0L0ERW2	Anti-sigma-28 factor FlgM
	+2.91	A0A0L0EUK7	Twitching motility protein PilT
	+3.04	A0A5S3V634	Methyl-accepting chemotaxis protein
	+3.11	A0A5S3V3M4	Methyl-accepting chemotaxis protein
	+4.18	A0A5S3V3J7	Methyl-accepting chemotaxis protein
	+2.46	A0A5S3V0Y1	Methyl-accepting chemotaxis protein
	+2.75	A0A5S3V0M8	Methyl-accepting chemotaxis protein
	+2.12	A0A5S3V0H3	Methyl-accepting chemotaxis protein
	+2.34	A0A5S3UZH1	Methyl-accepting chemotaxis protein
	+3.91	A0A5S3UXP0	Methyl-accepting chemotaxis protein
	+3.08	A0A5S3UXF8	Methyl-accepting chemotaxis protein
	+2.82	A0A5S3UWG2	Methyl-accepting chemotaxis protein
	+4.11	A0A5S3UVB3	Methyl-accepting chemotaxis protein
	+5.61	A0A5S3UU56	Methyl-accepting chemotaxis protein

	+1.92	A0A5S3UTY6	Methyl-accepting chemotaxis protein
	+1.91	A0A5S3UTH7	Methyl-accepting chemotaxis protein
	+2.51	A0A5S3UTB3	Methyl-accepting chemotaxis protein
	+2.45	A0A5S3UR67	Methyl-accepting chemotaxis protein
	+3.76	A0A5S3URP8	Methyl-accepting chemotaxis protein
Cell division	+1.93	A0A5S3V4C6	Cell division ATP-binding protein FtsE
	+2.68	A0A5S3V008	Cell division coordinator CpoB
	+2.31	A0A0L0EW35	Cell division inhibitor MinD
	+3.17	A0A5S3UZ24	Cell division protein DamX
	+3.18	A0A0L0EVX6	Cell division protein FtsA
	+3.41	A0A5S3V4C7	Cell division protein FtsX
	+1.38	A0A5S3UW61	Cell division protein FtsX
	+2.71	A0A5S3V1E1	Cell division protein FtsZ
	+2.78	A0A0L0EWQ7	Cell division protein ZapB
	+2.80	A0A5S3UQ38	Cell division protein ZipA
	+2.40	A0A0F4QML1	Cell division topological specificity factor
Core metabolism	+1.70	A0A0L0EMS8	Methylmalonate-semialdehyde dehydrogenase
	+2.45	A0A0F4QGS7	Ribose-phosphate pyrophosphokinase
	+2.90	A0A0U2Y0N0	Shikimate kinase
	+2.18	A0A0U3IDX6	Tryptophan synthase beta chain
	+2.80	A0A0L0ESU1	S-adenosylmethionine synthase
	+2.81	A0A0L0EN60	Succinate--CoA ligase [ADP-forming] subunit beta
	+3.39	A0A0L0EX71	Phosphoribosylformylglycinamidine cyclo-ligase
	+2.04	A0A0L0ETQ3	CoA transferase subunit A
	+2.46	A0A0L0ENB5	Dihydrolipoyllysine-residue succinyltransferase component of 2-oxoglutarate dehydrogenase complex
	+3.28	A0A0L0EP99	3-ketoacyl-ACP reductase
Type II secretion	+2.41	A0A5S3UQN2	Type II secretion system core protein G
	+4.51	A0A5S3UT29	Type II secretion system protein F
	+2.69	A0A5S3US13	Type II secretion system protein GspC
	+2.11	A0A5S3UR30	Type II secretion system protein GspD
	+3.58	A0A5S3UQK8	Type II secretion system protein J
	+3.00	A0A5S3URY9	Type II secretion system protein K
	+1.88	A0A5S3UQK7	Type II secretion system protein L
	+1.90	A0A5S3UQZ6	Type II secretion system protein M
	+3.32	A0A5S3V4K1	Type IV pili twitching motility protein PilT OS
mannosidase	-5.55	A0A5S3URX4	Sugar hydrolase

-: downregulate; +; upregulate.

Table S4. Antibiotic sensitivity of *Pseudoalteromonas rubra* S4059 growth on MB agar plate.

+: sensitivity. -: insensitivity. NT: not tested				
Antibiotics	10μg/mL	30μg/mL	50μg/mL	100μg/mL
Ampicillin	NT	NT	-	+
Kanamycin	NT	NT	-	+
Chloramphenicol	-	+	+	NT
Erythromycin	+	+	+	NT
Gentamycin	NT	NT	-	+

Strains, plasmids and primers

Table S5. Strains and plasmids used in this study.

Strains/plasmids	Genotype or relevant characteristics	Reference or source
**Bacterial strains**		
Escherichia coli		
WM3064	thrB1004 pro thi rpsL hsdS lacZΔM15 RP4-1360 Δ(araBAD)567 ΔdapA1341::[erm pir], 37 ℃. Donner strains in conjuation.	[1]
GB *dir pir*116	An arabinose-inducible *ETγA* operon (full-length *recE*, *recT*, *redγ*, and *recA*), a copy-up *pir*116 gene. Host strain for constructing suicide plasmids with R6K replication origin.	[2]
TOP 10	F- *mcrA* Δ(*mrr-hsd*RMS-*mcr*BC) Φ80*lac*Z ΔM15 Δ *lac*X74 *rec*A1 *ara*D139 Δ(*araleu*)7697 *gal*U *gal*K *rps*L (StrR) *end*A1 *nup*G.	Invitrogen™
Pseudoalteromonas rubra		
S4059	Wild type strain, Isolated from seaweed.	[3]
ΔGH19	GH19 chitinase gene in-frame deletion mutant of *P. rubra* S4059 Δ*GH19*	This study
**Plasmids**		
pDM4	Suicide vector for targeted mutagenesis; *sac*B; *ori*R6K; Cm^R	[4]
pDM4 –del-*GH19*	The left arm and right arm DNA region of the GH19 chitinase gene were PCR amplified from *P. rubra* S4059 genome and cloned in the pDM4 plasmid by direct cloning	This study

Kan^R: Kanamycin resistance; Cm^R: Chloramphenicol resistance

Table S6. Primers used in this study.

Primer	Sequence	Description	Expected size (bp)
GH19-L-F	GCtctagaACTCAATAATCCACTAAAGCC	Left arm of GH19	1032
GH19-L-R	ATTAAAGCCTAAAAAAGGACATCCTTTACATGTTG		
GH19-R-F	TGTAAAGGATGTCCTTTTTTAGGCTTTAATTTTACTTCG	Right arm of GH19	1014
GH19-R-R	CCGctcgagAGGGTTCCTTTGTACTTAAAC		
Cm^R F	GGCATTTCAGTCAGTTGCTC	Detection of Cm^R gene	525
Cm^R R	CCATCACAAACGGCATGATG		
GH19-pJet1.2-F	CCACATGTGGAATTGTGAGCTAAAGCCGCCACCAATGATG	GH19 homologous fragments	1914
GH19-pJet1.2-R	CTTATCGATACCGTCGACCCGGCAGACCCAGAATAAGCG		
GH19-pDM4-F	CATCATTGGTGGCGGCTTTAGCTCACAATTCCACATGTGG	pDM4 plasmid (GH19)	7054
GH19-pDM4-R	CGCTTATTCTGGGTCTGCCGGGTCGACGGTATCGATAAG		
GH19-p 1	GGGTTCCTTTGTACTTAAAC	Confirmation of GH19 mutants	1st: P1, P4 2452[a]; 1012[b]
GH19-p 2	CTTCAAGGACACATACCTTC		P2, P3 2577[a] bp; 1137[b] bp
GH19-p 3	CTATTCAGGCTACCAAAGC		2nd: P1, P2 3487[a] bp; 2047[b] bp
GH19-p 4	CAACAACATGTAAAGGATGTC		

[a] the size of WT; [b] the size of mutant.

Table S7. The normalized protein abundance of chitinolytic enzymes in S4059.

Accession	Description	chi-WT cells 1	chi-WT cells 2	chi-WT cells 3	chi WT sup 1	chi WT sup 2	chi WT sup 3	man-WT cells 1	man-WT cells 2	man-WT cells 3	manWT sup 1	man WT sup 2	man WT sup 3
A0A5S3USE2	GH18	32.06327	32.61660578	33.4121	37.44231	37.65211	37.49193	20.48135	20.43851	22.67027	28.92823	24.77495	25.07364
A0A5S3V351	GH18	20.6461	20.96568668	20.22428	23.46404	23.16674	23.25328	NA	NA	NA	NA	NA	NA
A0A5S3V0U4	GH18	28.15098	27.67043822	29.01734	34.34522	34.25175	33.45857	NA	NA	NA	NA	NA	NA
A0A5S3V6T3	GH18	26.33148	26.75637076	28.43113	32.76361	32.03789	31.64236	18.73021	NA	21.37777	26.46891	26.48377	26.36123
A0A5S3V3K3	GH18	27.80207	28.00121752	30.02482	36.11999	35.99521	35.4816	23.64874	24.07021	23.43214	28.80977	29.00245	28.72435
A0A5S3USH6	GH18	30.77192	30.92668926	32.04919	37.43495	37.65743	37.04639	NA	17.87567	NA	23.19466	23.32522	23.6994
A0A5S3V378	GH18	21.42957	21.86235625	21.93963	27.15255	26.57559	26.68415	NA	NA	NA	NA	NA	NA
A0A5S3UX38	GH19	19.85311	19.99490452	20.16807	26.66788	26.37509	22.88288	NA	NA	NA	NA	NA	NA
A0A5S3V1X9	GH20	28.35271	27.9787318	28.45681	24.3699	23.91617	25.1039	21.347	21.34887	21.51012	21.78837	NA	18.85425
A0A5S3UX95	GH20	28.62493	28.18178963	29.02698	27.56267	27.05506	26.8132	27.99074	27.8446	27.78883	18.63304	21.84458	24.56435
A0A5S3UV09	GH20	21.22181	21.59476677	22.67991	NA	NA	NA	NA	NA	NA	NA	NA	NA
A0A5S3UTD1	AA10	25.19116	25.3838232	27.22621	31.44592	31.29765	30.12035	NA	NA	21.18536	21.92399	21.03666	NA

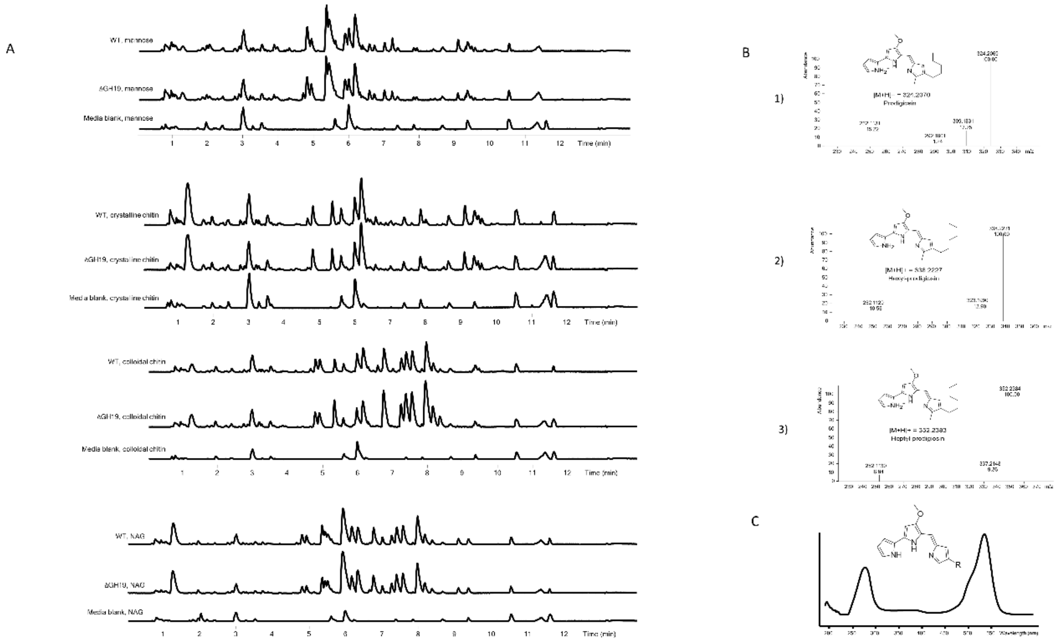

Figure S1. (A) Base peak chromatograms of *Pseudoalteromonas rubra* S4059 wild type (WT) and *ΔGH19* mutant when cultivated on marine minimal medium using mannose, crystalline chitin, colloidal chitin or N-acetyl glucosamine (NAG) as the carbon source. The red pigment prodigiosin and two of its analogues (hexyl prodigiosin and heptyl prodigiosin) could be identified in the extract and confirmed based on MS/MS experiments and the acquired absorption spectra. Experiments were done in biological triplicates, and a reference chromatogram of the sterile growth medium is included to show media components also present in the experiments. (B) Tandem MS spectra recorded for 1) prodigiosin, 2) hexyl-prodigiosin, and 3) heptyl-prodigiosin. The prodigiosin MS/MS spectra matches our in-house MS/MS library, and the characteristic fragment m/z 252.11, and loss of CH_3 (m/z 15.02) additionally matches those previously reported for prodigiosin and analogues [5]. (C) Recorded UV-Vis absorption spectrum of prodigiosin. All proposed prodigiosins share identical absorption spectra. The spetrum is in agreement with previously reported literature [6].

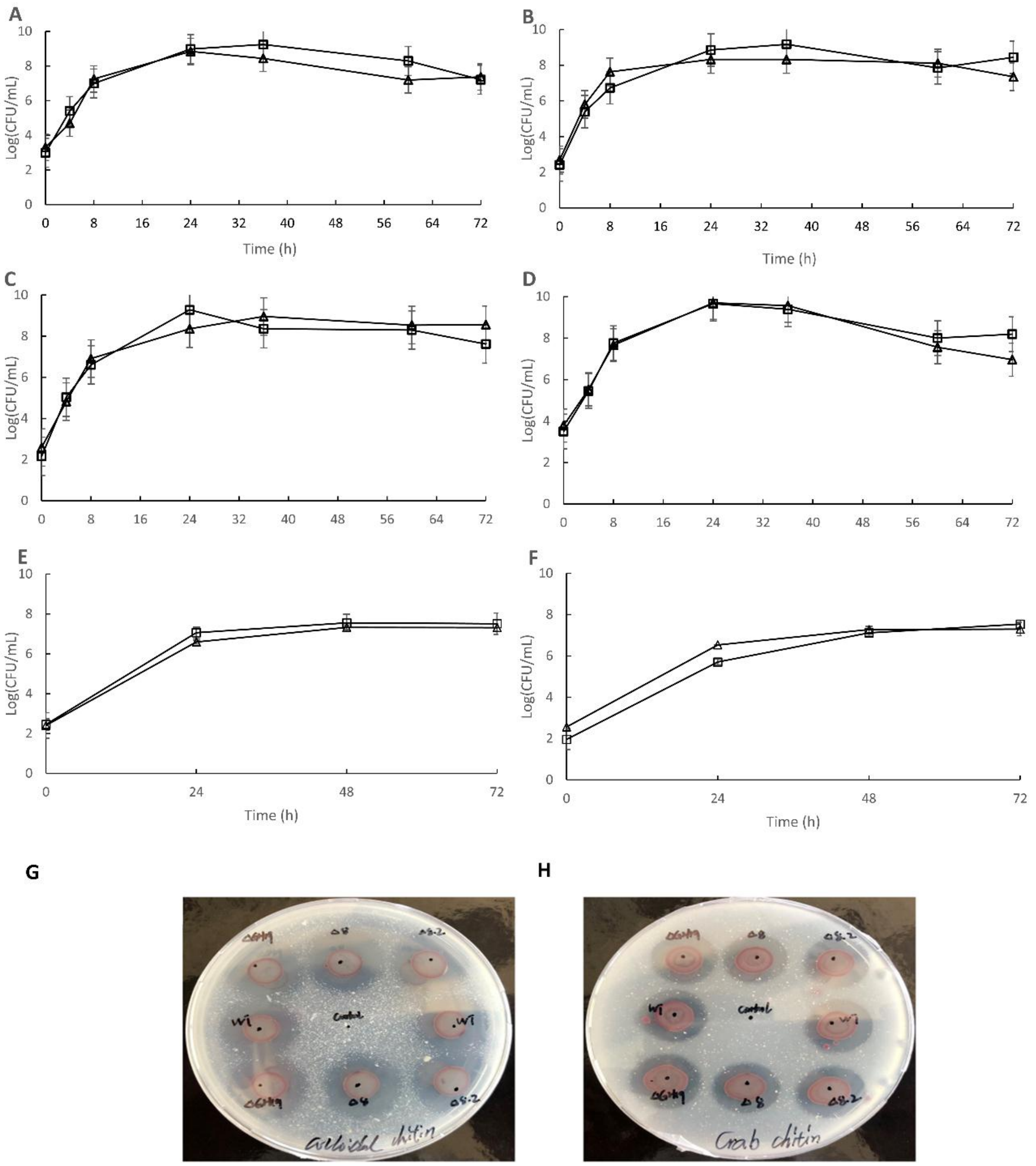

Figure S2. (A-D) Growth kinetics of *Pseudoalteromonas rubra* S4059 wild type (WT) and *ΔGH19* mutant in mannose (A), colloidal chitin (B), crystalline chitin (crab chitin) (C) and NAG (chitin monomer) (D) containing MMM with casamino acids. (E-F) WT and *ΔGH19* growth in crystalline chitin (E) or colloidal chitin containing MMM without casamino acid. Square: WT; Triangle: *ΔGH19*. The points are bio-replicates and error bars are standard deviation. (G-H) Chitin degradation activities of wild type and the mutant on colloidal chitin plate (G) and crystalline chitin plate (H).

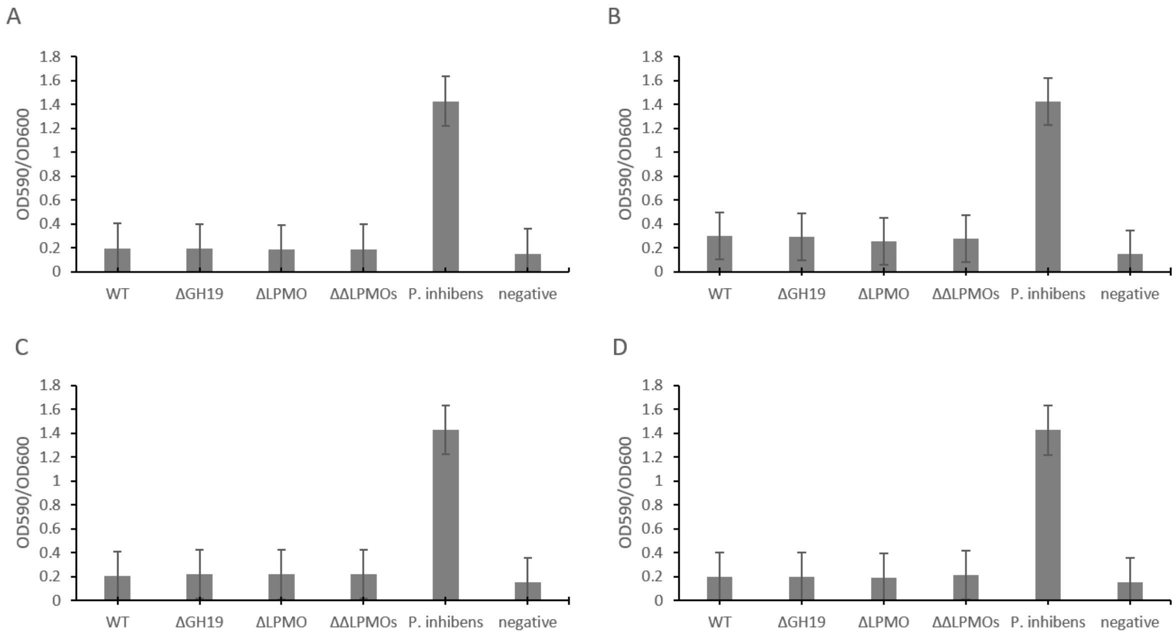

Figure S3. Biofilm formation on the microtiter-well plastic surface of *Pseudoalteromonas rubra* S4059 wild type and mutants in four different sole carbon contained medium as determined by the O'Toole & Kolter crystal violet assay. (A) in mannose; (B) in NAG (chitin monomer); (C) in colloidal chitin; (D) in crab chitin. Positive control is *Phaeobacter inhibens*; negative control is sterile media. Each experiments are repeated in bio-triplicates and error bars are standard deviation.

SUPPLEMENTARY EXPERIMENTAL PROCEDUR

Antibiotic sensitivity assay for selection marker. The antibiotic sensitivity of *P. rubra* S4059 to five antibiotics was tested: Ampicillin (Sigma, A9518), Kanamycin (Sigma, K4000), Chloramphenicol (Sigma, C0378), Erythromycin (Sigma, E6376), Gentamycin (Sigma, G3632). *P. rubra* S4059 was grown in MB at 25 °C to late exponential phase and a 10-fold dilution series plated on MA plates with antibiotics in the final concentration of 10 µg/mL, 30 µg/mL, 50 µg/mL, 100 µg/mL. The plates were incubated at 25 °C for 48 h and inspected for colonies. The assay was performed in triplicate.

Electroporation of *E. coli* strains. The electroporation procedure was modified from Wang *et al* [2]. Briefly, a pre-culture was grown and diluted 100 times in 5 mL fresh LB and grown to OD_{600}≈0.4. 1.5 mL cells were harvested at 6,000xg for 1 min. The cells were washed twice in 1 mL MilliQ H_20 and harvested at 6,000xg for 1 min. Around 200 ng DNA was added to the harvested cells and MilliQ H_20 was added to a final volume of 30 µL. The mixture was placed in a 1-mm electroporation cuvette and electroporated at 1,200 V. Cells were resuspended in 1 mL LB Broth without antibiotics immediately after electroporation and incubated in a 1.5-mL Eppendorf® thermomixer comfort at 37 °C, 750 rpm for 1 h. The recovered cells were spread on selection plates with antibiotics and incubated at 37 °C, overnight.

Chitin degradation. The protocol was modified from Paulsen *et al.* [7]. The medium consisted of 2% sea salt (Sigma, S9883), 1.5% agar, 0.3% casamino acid and 0.2% chitin (crystalline chitin from crab and colloidal chitin). Briefly, all strains (wild type and Δ*GH19* mutant) were grown in MB at 25 °C, overnight. The pre-cultures were diluted 100 times in 5 mL fresh MB and grown to OD_{600}=0.1. Twenty-µL culture was spotted on chitin plates. The plates were incubated at 25 °C for 7 days. A qualitative grading of chitinase activity was measured as previous description [7].

Biofilm formation assay. Biofilm formation was evaluated in 96-well polystyrene plates (Thermo scientific, 163320) with crystal violet staining as previous reported [8]. Cells were incubated with 5 mL MB in 50 mL tubes at 25 °C, 200 rpm overnight. The overnight cultures were diluted to OD_{600}=0.01 in MMM with different carbon sources (the same medium used for growth kinetics) in a volume of 0.5 mL. To establish the biofilm, 100 µL liquid from the diluted culture were added into each well in triplicate. The plates were incubated at 25 °C for 72 hours in a humidity chamber. The biofilm was visualized by crystal violet staining and was dissolved in 95% ethanol to measure the absorbance at 590 nm. *Phaeobacter inbibens* DSM17395 was used as positive control and fresh medium was used as negative control.

Anti-fungal activity assay. Anti-fungal activity assay was done with a modified protocol from Paulsen *et al*. 2016 [7]. In brief, *Aspergillus niger* (IBT 32191) was precultured from IBT Culture Collection at Department of Biotechnology and Biomedicine, Technical University of Denmark. A volume of 20 µL spore suspension of *A. niger* was added to a puncture well in the center of a MA plate. Then, 20 µL of overnight culture of *P. rubra* S4059 in MB was spotted 2 cm away from the center of well. Due to slow growth, we also tested that the spore suspension of *A. niger* was added 4 days prior to the S4059 culture. Plates from both settings were incubated and checked the size of inhibition zone every day for 14 days.

Preparation of desks from shrimp shells. Strip the shells off the shrimps carefully by hand and put it into 1L beaker with fresh dH_20. After striping all of shells, a teeth brush was used for cleaning the shrimp shells under flowing water to make sure no tissues (or meat) left on the shell. Then put the cleaned shrimp shells into a new 1L beaker with fresh dH_20 for washing. Tear the shrimp shells and use the puncher machine to punch the shrimp shells out. Remember each piece of shrimp shell should only be used for punching once and only pick the perfect circular shrimp shells (do not use the gaped shells or broken shells). Put the punched shrimp shells into 100 mL bottles with 30 mL, 3% Sea salts (Sigma, catalog number S9883). Do not let them dry. Otherwise, they will break easily. The shells in 3% Sea salts solution were autoclaved at 121 ℃ for 15 min and stored at 4 ℃.

References

1. Dehio, C.; Meyer, M. Maintenance of broad-host-range incompatibility group P and group Q plasmids and transposition of Tn5 in Bartonella henselae following conjugal plasmid transfer from Eescherichia coli. *J. Bacteriol.* **1997**, *179*, 538–540, doi:10.1128/jb.179.2.538-540.1997.

2. Wang, H.; Li, Z.; Jia, R.; Hou, Y.; Yin, J.; Bian, X.; Li, A.; Müller, R.; Stewart, A.F.; Fu, J.; et al. RecET direct cloning and Redαβ recombineering of biosynthetic gene clusters, large operons or single genes for heterologous expression. *Nat. Protoc.* **2016**, *11*, 1175–1190, doi:10.1038/nprot.2016.054.

3. Gram, L.; Melchiorsen, J.; Bruhn, J.B. Antibacterial activity of marine culturable bacteria collected from a global sampling of ocean surface waters and surface swabs of marine organisms. *Mar. Biotechnol.* **2010**, *12*, 439–451, doi:10.1007/s10126-009-9233-y.

4. Milton, D.L.; O'Toole, R.; Hörstedt, P.; Wolf-Watz, H. Flagellin A is essential for the virulence of Vibrio anguillarum. *J. Bacteriol.* **1996**, *178*, 1310–1319, doi:10.1128/jb.178.5.1310-1319.1996.

5. Lee, J.S.; Kim, Y.S.; Park, S.; Kim, J.; Kang, S.J.; Lee, M.H.; Ryu, S.; Choi, J.M.; Oh, T.K.; Yoon, J.H. Exceptional production of both prodigiosin and cycloprodigiosin as major metabolic constituents by a novel marine bacterium, Zooshikella rubidus S1-1. *Appl. Environ. Microbiol.* **2011**, *77*, 4967–4973, doi:10.1128/AEM.01986-10.

6. Couturier, M.; Bhalara, H.D.; Chawrai, S.R.; Monson, R.; Williamson, N.R.; Salmond, G.P.C.; Leeper, F.J. Substrate Flexibility of the Flavin-Dependent Dihydropyrrole Oxidases PigB and HapB Involved in Antibiotic Prodigiosin Biosynthesis. *ChemBioChem* **2020**, *21*, 523–530, doi:10.1002/cbic.201900424.

7. Paulsen, S.S.; Andersen, B.; Gram, L.; MacHado, H. Biological potential of chitinolytic marine bacteria. *Mar. Drugs* **2016**, *14*, doi:10.3390/md14120230.

8. O'Toole, G.; Kolter, R. Initiation of biofilm formation in Pseudomonas fluorescens WCS365. *Mol. Microbiol.* **1998**, *28*, 449–461.
